# The neurobiology of collective action

**DOI:** 10.3389/fnins.2013.00211

**Published:** 2013-11-19

**Authors:** Paul J. Zak, Jorge A. Barraza

**Affiliations:** ^1^Center for Neuroeconomics Studies, Claremont Graduate UniversityClaremont, CA, USA; ^2^Department of Neurology, Loma Linda University Medical CenterLoma Linda, CA, USA

**Keywords:** oxytocin, prosocial behavior, neuroscience, economics, empathy

## Abstract

This essay introduces a neurologically-informed mathematical model of collective action (CA) that reveals the role for empathy and distress in motivating costly helping behaviors. We report three direct tests of model with a key focus on the neuropeptide oxytocin as well as a variety of indirect tests. These studies, from our lab and other researchers, show support for the model. Our findings indicate that empathic concern, via the brain's release of oxytocin, is a trigger for CA. We discuss the implications from this model for our understanding why human beings engage in costly CA.

## Introduction

How do people come together to achieve a common goal? This essay will argue that the physiologic drivers of collective action (CA) are the same mechanisms that are involved in the experience of empathy. Specifically, we present a formal model and describe neuroeconomics studies from our lab that have revealed empathy, and empathic concern in particular, as a crucial component of CA. Herein we review studies from our lab that demonstrate the neuroactive hormone oxytocin instantiates empathy and promotes prosocial behaviors, including CA (for other similar reviews of the human oxytocin literature see Bartz et al., 2011; De Dreu, 2012; Feldman, 2012; Guastella and MacLeod, 2012; Kumsta and Heinrichs, 2012; Van IJzendoorn and Bakermans-Kranenburg, 2012; Carter, 2013; for similar reviews focusing on neural activity see Shamay-Tsoory, 2011; Decety et al., 2012). We begin with the understanding that most CA is not done for purely altruistic or other-regarding motives. For instance, people may volunteer for a cause out of concern for others, but may also volunteer out of a felt or social obligation, to build their reputation, or to feel better about themselves (e.g., Omoto and Snyder, 1995). This review focuses on the role of one particular motive for CA: empathy. A biologically based human capacity, empathy has been found to motivate prosocial behaviors (e.g., Eisenberg and Fabes, 1990; Batson and Oleson, 1991; Penner et al., 2005). Empathy can promote CA by reducing self-regarding concerns and enhancing other regarding motives (e.g., Batson, 1991). We propose that empathy is a motive for CA, an adaptive human behavior with neurobiological underpinnings (for similar arguments see Brown and Brown, 2006; de Waal, 2008; Gonzalez-Liencres et al., 2013).

This idea was captured in Adam Smith's (1759) masterwork The Theory of Moral Sentiments where he wrote, “Generosity, humanity, kindness, compassion, mutual friendship and esteem… please the indifferent spectator upon almost every occasion. His sympathy with the person who feels those passions, exactly coincides with his concern for the person who is the object of them” (Vol. 1, ch. iv, para. 313). In discussing sympathy, or “fellow-feeling” as Smith defined it, we will use the word *empathy* (a term derived from an 1858 coinage einfühlung or “feeling into” by German philosopher Rudolf Lotze (1817−1881) that more closely captures the notion of an innate human capacity for one individual to respond to the experiences of another (Davis, 1996).

The literatures describing empathy are large and diverse (Batson, 2010), but our focus is on a narrower notion, empathic concern. Empathic concern is an emotion that is felt *for* another person (also see Barraza and Zak, 2013) and has been called the “root of all altruism” (McDougall, 1926). Empathic concern has been used interchangeably with notions of compassion (Batson, 2010), though we prefer the former term as being less generally used and thus less prone to misuse. Those who become aware of distress in others and are able to regulate the arousal that arises from it are more likely to experience empathic concern (Eisenberg and Fabes, 1990).

**Figure 1:**
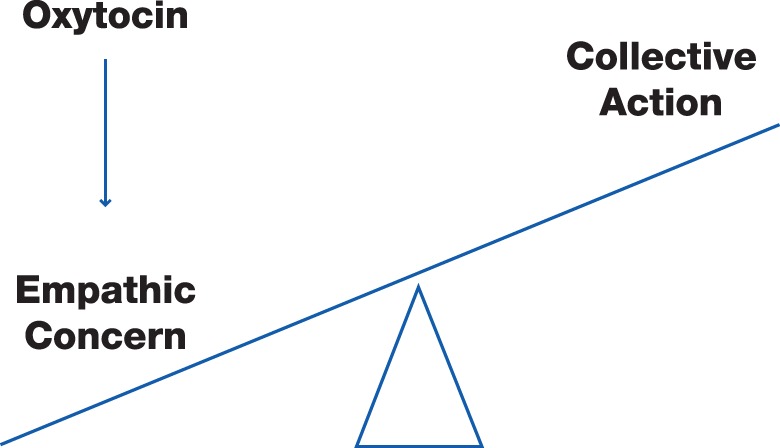
**A physiologic model of collective action.** Oxytocin induces empathic concern that increases the likelihood of collective action.

We begin by presenting a rationale for CA. Next, we introduce a neurobiologically-based model of prosocial behaviors in order to identify empathic concern as a proximal mechanism for CA. We then introduce evidence from recent studies from our lab suggesting a role for the neuropeptide oxytocin in producing empathic concern and inducing CA. Figure 1 summarizes the proposed relationships.

## A mathematical model of collective action

CA refers to a set of behaviors that are performed with others to meet a goal or strive to make progress on a desired outcome. CA includes both cooperative behaviors (where two or more people work toward a mutually beneficial outcome) and collective helping behaviors (where two or more people work for the benefit of others not involved in the action). CA can be a single event (e.g., assisting someone who is drowning, pitching in money or time for a group picnic) or can extend over a long period of time (e.g., volunteering weekends at a retirement home, or the provision of public goods). Thus, CA includes a wide array of actions that are done for the benefit of others at some cost to the individual, whether or not these benefits extend to the self.

Why do people intentionally engage in behavior where the self bears a direct or opportunity cost? Game theoretic models derived from the prisoner's dilemma show that conditional cooperation is typically a better long-term strategy than consistent defection (Axelrod, 1984). These models, however, generally focus on why people would engage in behaviors that, although benefiting others, eventually benefit the actor. Tellingly, some forms of CA may provide little or no direct, immediate, or guaranteed benefit to the actor (Melis and Semmann, 2010).

Empathic concern for another's welfare may be a proximate mechanism motivating individuals to engage in costly CA. Empathic concern is a candidate mechanism for CA because it allows individuals to focus on the state of others, even in situations where there may be no direct benefit for the actor (de Waal, 2008). For example, empathic concern after a signal of distress or request for help, resolves the problem of reciprocal motives for CA where the actor benefits at a later time by placing weight on the well-being of others.

Behavioral scientists have found that empathic concern tips the scale in favor for prosocial engagement (e.g., Batson, 1991; Davis, 1996; Sober and Wilson, 1998; Preston and de Waal, 2002). The arousal: Cost-reward model of helping behavior (Dovidio, 1984; Dovidio et al., 1991) states that in order for people to be motivated to help others, they have to first become aware of the need of others for help. Aversive arousal elicited through emotional contagion makes the need for intervention salient. Aversive arousal then motivates a cognitive weighing of the costs and benefits for acting prosocially. Empathic concern is assumed to increase the costs for not engaging, for example, producing guilt, shame, and further distress if the observer does not help or cooperate. An explicit model of prosocial emotions such as guilt and shame prompting costly prosocial behavior was proposed by Bowles and Gintis (2003). Empathic concern may reward those who help others, for example, producing a so-called warm glow utility flow (positive affect for engaging in helping others; Andreoni, 1990) or other internal reward (Harbaugh et al., 2007) as we will propose in the model below.

The empathy-altruism hypothesis (e.g., Batson, 1991; Batson and Oleson, 1991), suggests that an empathic response is a necessary component in human prosocial behaviors. The arousal experienced from witnessing another's aversive state leads to divergent affective reactions, especially distress and empathic concern. Whereas distress (self-focused aversive feelings) motivates a desire to reduce aversive arousal, empathic concern causes one to attend to the other's aversive state. Those who are distressed may seek to escape the arousing situation (either psychologically or physically) when it is less costly than staying involved (Batson, 1987). On the other hand, empathizing with those requiring help makes it difficult to disengage without seeking to relieve the other's distress.

A large number of psychological studies have supported the link between empathic concern and prosocial engagement. Instead of reviewing this extensive literature (e.g., see Davis, 1996; de Waal, 2008; Batson, 2010), we use volunteerism to illustrate the role of empathic concern in CA. Volunteerism is a form of CA that occurs in the context of groups and organizations, where people give of their time for the benefit of a person, group, or cause (e.g., Penner et al., 2005). Volunteerism is interesting because it is long-term planned behavior (Penner, 2002). As such, volunteering is less influenced by situational factors than other prosocial actions. Further, volunteering is typically focused on aiding strangers to whom there is no social obligation (Omoto and Snyder, 1995). In general, volunteers have been found to be more dispositionally empathic than non-volunteers (e.g., Rushton, 1984; Bekkers, 2005). Those who score high in dispositional empathy anticipate feelings of empathy and satisfaction during volunteering and are more willing to volunteer because of those feelings (Davis et al., 1999). Individuals who report empathy-driven prosocial motives for volunteering, for example expressing values and concern for their community, are found to persist longer as volunteers than those who endorse self-oriented motives like enhancing their employability or to feel better about themselves (e.g., Clary and Orenstein, 1991; Penner and Finkelstein, 1998). These findings indicate that empathic concern is a key factor in motivating and sustaining one form of CA—volunteerism. In the model of CA that follows, we seek to clarify the mechanisms through which empathic concern and distress affect other-regarding behaviors.

The model we propose is a neurologically-informed extension of the model in Zak et al. (2007) that is based on a decade's worth of experiments using an inductive approach (Park and Zak, 2004; Vercoe and Zak, 2010) in which experimental treatments are systematically varied before a model is proposed. The goal in presenting this model is not to replace traditional game theoretic models of CA, but to extend these models to include the role of empathic concern during social interactions.

The model takes as its foundation a model introduced in a footnote by the prominent Irish social philosopher Edgeworth (1881/2012) in his book *Mathematical Psychics: An Essay on the Application of Mathematics to the Moral Sciences* where utility is obtained from one's own consumption and a weighted utility of another's consumption (Edgeworth, 1881/1967). Andreoni (1990); Sally (2001, 2002), and Levitt and List (2007) have proposed similar models without drawing on neural findings, while Morishima et al. (2012), develop a neurally-informed mathematical model based on theory of mind. Similar to Morishima et al. we propose a model steeped in experimental findings that can shed new insights into CA. The model differs from Edgeworth and the existing literature by including responses that are conditional on one's own, and the other's, physiologic states.

The decision-maker, who we will identify as person 1, faces the following decision problem

$$\begin{array}{l} {\text{Max}}_{b1\;b2}E\{U(b_{1})+\alpha (\tau )U(b_{2})\}\\ \;s.t.\;b_{1}+b_{2}=M \end{array}$$

where *U*(*b*_1_) is the utility person 1 receives from consuming benefits *b*_1_, *b*_2_ is the benefit that person 2 receives from person 1, *U*(*b*_2_) is the utility person 2 obtains from *b*_2_, and total resources, *M*, are finite. Assume *U*(*b*) is increasing, continuous and strictly concave. Person 1 chooses *b*_1_ and *b*_2_ through this constrained optimization problem. We will call this the Empathy-Collective Action model.

Edgeworth called the weight α on the other's utility “effective sympathy” (1881/1967, p. 53) and considered it a constant; using Lotze's definition of emotional contagion, we will call α “empathic concern.” Our Empathy-Collective Action model generalizes Edgeworth by identifying CA as an individually costly behavior and by taking into account the motivation for prosocial action by letting empathic concern depend on the situation the decision-maker faces. Specifically, let α(τ): [0,1]→ **ℜ**^+^ be a continuous hyperbolic function where empathic concern, α, depends on the observed distress of person 2, τ. The parameter τ captures the distress that motivates the decision-maker to pay attention to the needs of the other person. As previously discussed, “distress” should be understood as any situation in which the behavior or emotional state of another (or group of others) suggests that they may need assistance. The function α has the following properties, α(0)≥0, lim_τ→∞_ α(τ) = 0, and τ^*^ = argmax α(τ), with α(τ^*^) > α(0), and τ^*^ finite. That is, α(τ) has the shape of a parabola.

The empathic concern function α(τ) is hyperbolic because moderate distress motivates action, but high degrees of distress are aversive causing one to want to escape rather than help (e.g., Batson et al., 1987). For example, if one sees someone sprain an ankle and fall to the ground, most people are motivated to help. Seeing someone with a bloody compound fracture of the ankle may be so distressing that many bystanders will flee and avoid helping. Alternatively, distress may arise from social pressures of inaction.

In the Empathy-Collective Action model, when α(τ) = 0, person 1 is completely self-interested, and when α(τ) = 1 s/he is other-regarding, sharing benefits equally with person 2. Values of α(τ) > 1 cause person 1, at an optimum, to offer more resources to person 2 than she keeps herself. It is straightforward to prove that as α rises, the benefits to person 2, *b*_2_, increase. Different values of α would account individual variations in empathic concern and resulting differences in individually-costly CA. Indeed, CA, where an individual bears a direct or opportunity cost during CA, requires a positive value of α(τ). The model's value is that is shows how individual variations in empathic concern (α) and the social environment (τ) can be included in a game-theoretic model of CA. If one exhibits low CA in a given situation, the model predicts that either empathic concern or one's perception of the needs of others (or both) is low. For example, an adult waiting to cross a busy street may not elicit costly CA by those nearby, but a small child alone seeking to cross such a street is likely to produce greater CA, especially among parents who may be more sensitized to children.

Our next task is to present neurobiological evidence showing that empathy affects CA.

## Neurobiological mechanisms

Knowing the neurobiology of empathic concern not only provides additional information on mechanism, but may also produce additional testable implications and applications (see Neurobiological Mechanisms). A large body of work now exists on the neural basis for empathy using functional MRI which have been reviewed in detail elsewhere (see Lamm et al., 2011; Shamay-Tsoory, 2011; Bernhardt and Singer, 2012). These studies generally locate empathy within the brain's pain matrix, specifically in the anterior cingulate cortex and the anterior insula (Singer et al., 2004, 2006; Hein and Singer, 2008). However, these studies focus on the distress aspect of social engagement by studying responses to pain rather than the possible rewards of empathic concern.

The Empathy-Collective Action model of prosocial behavior that posits a utility flow or “warm glow” is consistent with findings from two studies using fMRI by examining donations to charities. Moll et al. (2006) found that brain regions differentially more active during donations to preferred charities compared to unpreferred charities included striatal regions associated with rewarding stimuli. These researchers also found that contrasting brain activity during charitable donations and individual reward revealed activation in the subgenual cortex, a brain region that modulates rewards associated with affiliative behaviors. In a related study of charitable donations, Harbaugh et al. (2007) found that donating to a charity, relative to keeping money for oneself, also produced activation in striatal regions of the brain. They further showed that voluntary donations to charity were associated with a greater subjective experience of satisfaction and larger striatal activation than mandatory donations.

### The role of oxytocin

The best evidence for the role of empathic concern affecting CA would be to discover a manipulable neural mechanism that would raise or lower α in the Empathy-Collective Action model. The word “manipulable” is important here to demonstrate that such a mechanism directly *causes* CA. If we push on this mechanism (somehow), we would expect to see less self-focused benefits *b*_1_, and more other-focused benefits *b*_2_.

Oxytocin (OT) is an evolutionarily ancient molecule that is a key part of the mammalian attachment system supporting costly care for offspring. In socially monogamous mammals, OT and a closely related hormone, arginine vasopressin, facilitate attachment to and protection of mates (see Carter, 1998). Maternal (and in some species paternal) care for offspring is a template for more general other-regarding behaviors (Sober and Wilson, 1998; de Waal, 2008). In the human brain, high densities of OT receptors are primarily found in the amygdala, hypothalamus, and subgenual cortex (Tribollet et al., 1992; Barberis and Tribollet, 1996), brain regions associated with emotions and social behaviors.

OT can be measured in blood and cerebral spinal fluid, and synthetic OT can be infused into human beings intravenously or intranasally to gauge its effects on behaviors (Churchland and Winkielman, 2012). A key issue for studying OT in humans is that under physiologic stress, central (brain) and peripheral (body) OT co-release (Wotjak et al., 1998; Neumann, 2008). This means that a change in blood levels in OT after a stimulus is likely to be positively correlated with changes in OT in the brain. In addition, peripheral OT binds to receptors in the heart and vagus nerve, reducing anxiety and cardiovascular tone (see Porges, 2001, 2007) and thereby signaling approachability. OT binding in animals is associated with the modulation of midbrain dopamine and serotonin (Pfister and Muir, 1989; Liu and Wang, 2003).

Studies using OT infusion in humans have shown that it enhances the ability to infer others' emotions and intentions from facial expressions (Domes et al., 2007). OT also increases the time spent gazing toward the eye region of the face (Guastella et al., 2008), and the recognition of faces (Savaskan et al., 2008). Mice with the gene for the OT receptor knocked out have social amnesia—they do not appear to remember animals they have previously encountered (Ferguson et al., 2000).

Situations that motivate CA often involve a request for help. Such requests may provoke both empathic distress and concern as in the Empathy-Collective Action model. OT infusion has been shown to reduce activity in the amygdala in response to socially fearful stimuli (Kirsch et al., 2005) and fear conditioned stimuli (Petrovic et al., 2008). By reducing anxiety, OT may help people sustain CA over extended periods of time. Social psychologist Shelley Taylor calls this the “tend and befriend” role of OT (Taylor et al., 2000; Taylor, 2006), where OT reduces anxiety and promotes affiliative behaviors in response to stress.

### Trust, reciprocity, and cooperation

Our lab was the first to demonstrate that OT promotes prosocial behaviors among human beings (Zak et al., 2004, 2005). We began this research in 2001 by examining the role of OT in facilitating trust between strangers. In these studies, we used a task from experimental economics called the trust game (Berg et al., 1995). In our trust experiments, participants were endowed with $10 to compensate them for their time and discomfort (see below). They were then given the opportunity to increase their earnings by making a single decision by computer and without coordinating with others using their $10. For this task, they were matched randomly in dyads with random assignment to the roles of decision-maker 1 (DM1) or decision-maker 2 (DM2). All DMs received extensive and identical instructions informing them that DM1 could transfer some of his or her endowment to the DM2 in dyad, and this amount would be removed from DM1's account and tripled in DM2's account. DM2 was then notified by computer of the tripled transfer from DM1 and was reminded of the total in his or her account. After this, the software prompted DM2 to return to DM1 any amount from zero to the account total. The return transfer was not tripled and was removed from DM2's account on a one-to-one basis. After these two decisions, the interaction was concluded. The consensus view in economics is that the DM1 transfer denotes trust, and the DM2 transfer captures reciprocity or trustworthiness.

So why would DM2 return any money, something participants do 98% of the time (Zak et al., 2007)? We found that the more money DM2s received, the greater the increase in OT. Importantly, the higher the spike of OT for DM2, the more she or he reciprocated by returning money to the DM1 who showed trust (Zak et al., 2004, 2005; Zak, 2012). Nine other hormones (e.g., vasopressin, estradiol) were ruled out for mediation or interactive effects, supporting the direct link between endogenous OT release and trustworthiness.

We next demonstrated the causal effect of OT on trust by administering 24IU of synthetic OT intranasally, a method utilized to enhance OT levels in the brain. After allowing for an hour for the OT to enter the brain, participants played the trust game. Not only did the average level of trust rise for those given OT, more than twice as many people on OT showed maximal trust by sending *all* of their money to a stranger (45 vs. 22% for those on placebo; Kosfeld et al., 2005). There was no effect of OT on an objective risk-taking task, providing evidence for its uniquely social effects. Moreover, the results were not due to changes in mood or cognitive blunting. These studies provide evidence that OT helps us determine who to trust and when to reciprocate, two key ingredients for CA.

Certainly trust can promote CA, but our trust research left open two important questions: are there non-pharmacologic ways to raise OT? and, is OT directly associated with empathic concern? In our trust experiments, the receipt of money denoting trust resulted in a substantial spike in endogenous OT relative to baseline. Prior to our work, the only known ways to raise OT in humans were to go into labor, to breastfeed a child, or to engage in sexual activity. These methods of raising OT are impractical for laboratory experiments, so we began to search for other ways endogenous OT might be manipulated. Research in rodents provided equivocal data that belly stroking might induce OT release. To test this in humans, we used licensed massage therapists to give participants a 15-min moderate pressure back massage. A control group simply rested quietly for 15 min on different days. Participants had their blood drawn and played the trust game one time. We found that massage raised OT (Morhenn et al., 2008, 2012), and for DM2s in the trust game, massage primed the brain to release 16% more OT than DM2 controls. Amazingly, reciprocation was 243% higher by DM2s in the massage group relative to DM2 controls (Morhenn et al., 2008). The change in OT strongly predicted the amount of money DM2s would sacrifice to reciprocate to DM1s.

We next undertook direct tests of the zero-sum Empathy-Collective Action model using a task called the Ultimatum Game (UG Güth et al., 1982). In this game, participants were again put into dyads and randomly assigned to the roles of DM1 and DM2. DM1 began the experiment with $10 while DM2 began with nothing. After extensive and identical instructions, DM1 was prompted by computer to propose a split of the $10 to DM2. If DM2 accepted the proposal, the money was paid. The catch was that if DM2 rejected the proposal, both DMs received nothing. In Western countries, offers less than $3 are nearly always rejected. We hypothesized that raising OT would increase empathy, α, and generate more generosity (generosity was defined as the amount a DM1 proposal exceeded the minimum acceptable offer by DM2s). Note that using the zero-sum UG, rather than a positive-sum trust game, sets the bar for the effects of OT substantially higher than in positive-sum games. In the trust game, we showed that OT was associated with reciprocity but that on average both DM1s and DM2s increased their earnings. In the UG OT was hypothesized to affect costly generosity in which more for DM2 meant less for DM1. This is just what we found. Infusing 40IU intranasally into participants caused an 80% increase in generosity relative to subjects who received a placebo (Zak et al., 2007). Generous participants left the lab with less money, but were not less happy on debriefing than those who were not generous. This provided the first evidence α could be manipulated by manipulating central OT.

The second test of the Empathy-Collective Action model used testosterone infusion to create “alpha males” in a double-blind cross-over paradigm (Zak et al., 2009). There is some evidence that testosterone inhibits OT binding to its receptor (Insel et al., 1993) and thus testosterone was expected to reduce generosity. This was indeed what we found. We raised total testosterone an average of 60% above baseline (free testosterone, and dihydrotestosterone, which are more active biologically than total testosterone, were raised 97 and 128% respectively; all changes were greater than zero at *p* < 1E-6). Men whose testosterone was artificially raised, compared to themselves on placebo, were 27% less generous in the UG. Moreover, the reduction in generosity fell rapidly as a man's level of total-, free- and dihydro-testosterone (DHT) rose, revealing a parametric effect of testosterone on generosity. For example, participants in the lowest decile of DHT had 85% higher average generosity ($3.65 out of $10) compared to generosity by those in the highest decile of DHT ($0.55 out of $10). Interestingly, the enhanced “alpha males” also had a 5% higher threshold (*p* = 0.001) to punish those who were ungenerous toward them. This experiment revealed that α could be reduced in the Empathy-Collective Action model.

In a third experiment, we examined whether endogenous OT was associated with the subjective experience of empathic concern by having participants watch a 100 s highly emotional video of a father and his son who has terminal brain cancer (Barraza and Zak, 2009). A control video had the same father and son going to the zoo but did not mention cancer or death. We found that watching the emotional video caused a 47% increase in OT relative to baseline. Importantly, the change in OT was correlated with subjective reports of empathic concern once we controlled for the distress that participants felt. We also found that those who were more empathically engaged made more generous offers in the UG, and generosity in the UG was associated with larger donations of participants' earnings to charity at the conclusion of the experiment. Participants who scored high in a measure of dispositional empathy (using the Interpersonal Reactivity Index, Davis, 1983), experienced greater empathic concern after the emotional video and had a larger increase in OT after viewing the emotional video. The participants who were most empathic and released the most OT were women; women were also more generous and gave more money to charity than did men. This study is the first to provide direct evidence that OT is associated with empathic concern, confirming the intuition of Adam Smith and the design of the Empathy-Collective Action model.

### Defectors and free-riders

Defection is the death-knell of CA. When people begin to free-ride, for example in public goods games, others typically follow suit (Camerer, 2003). In our studies using the trust game using college students, we find that 95% of DM2s who have been trusted reciprocate. The degree of reciprocation for this 95% are predicted by their OT levels. The other 5% are unconditional non-reciprocators, they return nothing or very little money no matter how much they are trusted. We found that OT levels of non-reciprocators are abnormally high, indicating OT dysregulation. Psychologically, these people have traits similar to psychopaths (Zak, 2005, 2012).

We have recently extended this finding by studying patients with social anxiety disorder (Hoge et al., 2008). They, too, have high levels of OT. Because the brain works through contrast, high OT masks any additional OT release when receiving a signal of trust, thus inhibiting a behavioral response. Similarly, a study of those diagnosed with borderline personality disorder (BPD), which is associated with a compromised ability to interpret social signals, showed an inability to maintain reciprocity in the trust game (King-Casas et al., 2008). This inability to cooperate seemed to be mediated by abnormal activity in the anterior insula, a brain region previously associated with empathy for pain (Singer et al., 2004, 2006); whereas psychologically healthy individuals showed a strong parametric relationship between amount received in the trust game and anterior insula activation, no such relationship was found for BPD subjects suggesting a possible empathy deficit in BPD.

Our discovery of the “five percent rule” for free-riders (Shermer, 2008; Zak, 2012) in a fixed institutional setting is important in understanding CA. It suggests that not all people can be expected to participate in a collective project, even when the issue is salient and people are highly motivated. When the social, economic or institutional environments are less than optimal, greater defection from CA will be expected as high levels of stress inhibit OT release (Carter, 1998). This is reflected in a low value of α in the Empathy-Generosity model, making the environment in which CA problems are solved important (Dietz et al., 2003). On the upside, our studies indicate that the majority of the population–including a study of aboriginal people in Papua New Guinea (Zak, 2012) release OT for a large variety of stimuli.

### Collective action through charitable institutions

We have now conducted several studies examining giving through charitable institutions. Charitable donations are unique from other forms of CA as it is typically done without any direct exposure to the beneficiary or direct knowledge of how the individual contributions will be used. Though performed by individuals, charitable giving functions through the collective contributions made to an institution to address an issue of interest to its contributors. Barraza et al. (2011) examined whether 40IU of OT would increase donations in a lab donation task. Participant in the OT condition gave 48% more money than those in the placebo condition. This result was later replicated by others using a smaller dose (24IU) and a different charity (Van IJzendoorn et al., 2011). In another study, participants viewed public service announcements (PSAs) relating to social and health related issues after 40IU of OT infusion (Lin et al., 2013). Participants were given an opportunity to donate some of their earnings to the charities promoted in the ads. We found those who received OT donated to 33% of the causes while participants receiving the placebo donated to 21% of the featured charities. OT also increased the size of donation by 56% compared to placebo.

Another set of evidence comes from a growing body of research examining the association between single nucleotide polymorphisms (SNPs) of the oxytocin receptor (OXTR) gene, and social behaviors. Work from others indicated an association between OXTR SNPs and empathy (Rodrigues et al., 2009; Wu et al., 2012a) as well as prosocial behaviors (Poulin et al., 2012; Wu et al., 2012b). In a recent study (Barraza et al., in preparation) we explored if OXTR SNPs affected CA done through charitable institutions. Three of the OXTR SNPs examined (rs237887, rs2268490, rs2254298) were linked with making a charitable contribution in a laboratory task. Participants were also asked to report their donations to charitable institutions outside the lab. Here, an association between OXTR and monetary donations was found for rs237887 (AA donating more than AG/GG), and rs53576 (AA/AG donating more than GG). Individuals with AA/AG genotype of rs53576 were found to be more likely to donate to religious charities (versus GG). Unexpectedly, we discovered that these same participants (rs53576: AA/AG) were more religious than their counterparts (rs53576: GG). Mediation analysis and indicated that the association between rs53576 and donations was a result of the relationship between rs53576 and religiosity. A possible interpretation is that OT may function by promoting CA through membership in an existing group.

### Ritual and intergroup behavior

CA involves both coordination with and a preference to affiliate with group members. It has been hypothesized that OT motivates cooperation especially for one's in-group by promoting (i) in-group favoritism, (ii) in-group cooperation, and (iii) defense-motivated non-cooperation toward threatening outsiders (De Dreu, 2012). OT administration increases bias for ones in-group when groups are formed for the experiment itself (De Dreu et al., 2010, 2011; Stallen et al., 2012). Although these studies provide evidence for in-group preference, they do not provide support for OT promoting antisociality toward an out-group (see Van IJzendoorn and Bakermans-Kranenburg, 2012) and may be alternatively explained by OT's social saliency properties (Chen et al., 2011). Moreover, OT's in-group-specific effects may only arise out of zero-sum tasks between groups, where cooperation can only be performed at a cost to an out-group. Support for this interpretation was found by Israel et al. (2012) using a task that allowed for intergroup cooperation. These scholars reported that OT promoted both in-group and out-group cooperation, although those who received OT allocated more resources benefiting their in-group compared to placebo recipients. We have produced results that fall somewhere in between the DeDreu et al. and Israel et al. studies. In our study of charitable donations mentioned above, we found OT increased the size of charitable donations with a trend toward a preference for an in-group vs. an out-group charity (American Red Cross or the Palestinian Red Crescent Society; Barraza et al., 2011). It appears that OT may promote in-group CA, but may also support CA across groups when there is a collective benefit available for everyone.

Our lab has recently examined a different question: why do naturally existing groups engage coordinated and costly ritualistic behaviors? Human life is replete with rituals and we hypothesized that rituals may induce the release of OT to reinforce group attachment. In this project (Terris et al., in preparation) we examined OT release before and after rituals for several secular and religious groups. Groups also made decisions in several economic tasks, [trust game (TG), ultimatum game (UG), and dictator game (DG)] by computer, with in-group and out-group members. We found that OT significantly increased for some groups after performing ritual (marching in unison, singing religious songs), but not for others (Christian prayer). We also observed a positive correlation between positive regard toward the in-group after the ritual and how much one gave to one's in-group relative to the out-group in the TG and DG, but not the UG. No association was observed between OT change induced by ritual and prosocial behavior toward in- or out-groups. These results indicate that although some rituals increase plasma OT, the increase does not appear to influence in-group preferences. This work suggests that OT can unite people to act as a group, but does not necessarily injure out-group collaboration when there are shared interests at stake.

### Trust in political institutions

Political actions, such as voting and campaigning, are another form of CA. Our lab has explored how OT administration affected trust in government officials and institutions during the 2007 Democratic and Republican primaries (Merolla et al., 2013). We found that participants given 40IU intranasal OT reported more agreement with the statement that most people can be trusted than those on placebo, especially when examining those low on pre-treatment interpersonal trust. Although OT did not directly impact trust in the government, we found Democrats on OT were more trusting of both Democrat and Republican politicians, and the federal government in general, when compared to those on placebo. When trust in government is higher, civic CA is likely to follow.

Generalized trust at the national level affects trust between individuals in the trust game (Holm and Danielson, 2005). Generalized trust levels strongly predict rates of economic growth in a cross-section of developed and less developed countries in part by facilitating CA (Zak and Knack, 2001). Generalized trust levels are also highly correlated with other forms of social capital such as paying taxes and other civic norms (Knack and Keefer, 1997), and trust and self-reported rates of happiness are very highly correlated at the country level (Zak and Fakhar, 2006) as are happiness levels and some forms of CA (e.g., volunteering; Post, 2005).

## Conclusion

Most traditional evolutionary and economic models do not attempt to provide proximate mechanisms to explain the wide array of behaviors that are called CA. These models have caused some behavioral scientists to erroneously conclude that costly prosocial behaviors are “irrational” or manipulative, presuming that individuals engaging in CA are hiding behind a “veneer” covering their true selfish instincts (e.g., de Waal, 2006). We presented a neurobiologically-informed model of individually-costly behaviors that benefit others. This model, with the hormone oxytocin at its core, accounts for physiologic factors that are not provided in extant models, particularly for the role of empathic concern. It is also consistent with experiments we have run that reveal substantial amounts of costly other-regarding behaviors, even in blinded one-shot depersonalized settings.

Those unfamiliar with the existing body of research on oxytocin may be left with the impression of OT as a purely prosocial hormone. This is not the case. OT has been implicated with behaviors that could be considered antisocial including ethnocentrism (De Dreu et al., 2011), envy (Shamay-Tsoory et al., 2009), and less adherence to fairness norms in certain contexts (Radke and De Bruijn, 2012). Moreover, there are methodological concerns about oxytocin administration (Churchland and Winkielman, 2012; Guastella et al., 2012), and peripheral oxytocin measurement (McCullough et al., 2013). The state of oxytocin research is still in it's infancy. The Empathy-Collective Action model seeks to take these disparate findings and provide a game theoretic structure to understand how OT affects human social behaviors.

The strength of our approach lies in integrating methodologies and evidence across disciplines (Zak, 2004). More generally, our research on the neuroeconomics of social behaviors has revealed that empathic concern serves as an internal compass that can result in CA (Zak, 2011). Adam Smith was right on target, fellow-feeling does appear to be the basis for many moral behaviors and CA. Research from our lab has simply identified a neurochemical mechanism behind Smith's intuition.

### Conflict of interest statement

The authors declare that the research was conducted in the absence of any commercial or financial relationships that could be construed as a potential conflict of interest.
